# Case study: risk associated to wearing silver or graphene nanoparticle-coated facemasks for protection against COVID-19

**DOI:** 10.1007/s00204-021-03187-w

**Published:** 2021-11-16

**Authors:** Carmen Estevan, Eugenio Vilanova, Miguel A. Sogorb

**Affiliations:** Instituto de Bioingeniería, Universidad Miguel Hernández de Elche, Avenida de la Universidad s/n, 03202 Elche, Spain

**Keywords:** Facemasks, Silver nanoparticles, Grapheme, Risk assessment, COVID-19

## Abstract

The world is living a pandemic situation derived from the worldwide spreading of SARS-CoV-2 virus causing COVID-19. Facemasks have proven to be one of the most effective prophylactic measures to avoid the infection that has made that wearing of facemasks has become mandatory in most of the developed countries. Silver and graphene nanoparticles have proven to have antimicrobial properties and are used as coating of these facemasks to increase the effectivity of the textile fibres. In the case of silver nanoparticles, we have estimated that in a real scenario the systemic (internal) exposure derived from wearing these silver nanoparticle facemasks would be between 7.0 × 10^–5^ and 2.8 × 10^–4^ mg/kg bw/day. In addition, we estimated conservative systemic no effect levels between 0.075 and 0.01 mg/kg bw/day. Therefore, we estimate that the chronic exposure to silver nanoparticles derived form facemasks wearing is safe. In the case of graphene, we detected important gaps in the database, especially regarding toxicokinetics, which prevents the derivation of a systemic no effect level. Nevertheless, the qualitative approach suggests that the risk of dermal repeated exposure to graphene is very low, or even negligible. We estimated that for both nanomaterials, the risk of skin sensitisation and genotoxicity is also negligible.

## Introduction

On December 31 2019, World Health Organization (WHO)^1^ released a note entitled “*Pneumonia of unknown cause reported to WHO China Office*”^2^ This initially called pneumonia was soon renamed as COVID-19 and is indeed an infectious disease caused by the SARS-CoV-2 virus (Zhu et al. 2020). The virus quickly spread worldwide and on March 11, 2020 WHO declared the COVID-19 outbreak a global pandemic (Cucinotta and Vanelli 2020). In the moment of draft this paper, the number of cases in the world was 235 million with 4.8 million of fatalities (Johns Hopkins Coronavirus Resource Center 2021). The virus has affected to both developed and non-developed countries, being the number of cases in both US and European Union countries of 44 and 37 million, respectively.

The virus spreads through airborne in small liquid particles expelled by infected people when they sneeze, cough, speak or breathe (Abd El-Wahab et al. 2020; Morawskaa and Caob 2020). This makes that one of the most effective prophylactic measures against the infection is wearing appropriate facemasks covering mouse and nose. Indeed, Tabatabaeizadeh (2020) found in a meta-analysis with four studies a relative risk of infection when wearing masks of 0.12. Eikenberry and co-workers (2020) modelled that an 80% adoption of moderately (50%) effective masks in the State of New York could reduce, in certain moment of the pandemic, the peak daily death rate by 34%–58%. Another review found a link between mouth and nose covering and relevant protection against the pathogen (Matuschek et al. 2020). All these evidences induced Governments to declare facemask-wearing mandatory in different spaces, especially in indoor areas.

Novel designs of respiratory protection equipment and facemasks include the coating of natural fibres initially used as filter barriers with certain nanomaterials (NMs), since the antimicrobial properties of several NMs was largely known (O’Dowd et al. 2020). Two of the NMs most widely used in facemasks are silver nanoparticles (NPs) (Ramaiah et al. 2021) and graphene (Srivastava et al. 2020).

Due to the above-stated considerations about the mandatory use of facemasks, since the outbreak of the virus to today there have been, and still there are, hundreds of millions of people regularly wearing facemasks. This massive use in general population and in occupational settings raised a concern as regard the safety of the facemasks coated with NMs. Indeed, the Canada Government released an announcement on May 2, 2021 alerting that facemasks that contain graphene may pose health risks; this announcement was updated on July 13 and on September 28 2021 with lists of model number of graphene-based authorised facemasks.^3^

Overall, the risk assessment associated to facemask wearing is strongly advisable. In this manuscript, we assess, based on publically available information and following methodologies used by European Regulatory Agencies, the potential risk for general population and workers of wearing facemasks coated with silver and graphene NMs. In the case of silver NPs, since the available information was found for oral administration and in the exposure scenario of our interest the route of exposure is dermal, we derived a systemic no effect level (DNEL). The derived systemic DNELs were several orders of magnitude higher than the estimated exposures; suggesting that, despite the uncertainties, the use of facemasks coated with silver NPs is safe. The lack of data made impossible to take a similar approach for graphene NMs, although a qualitative risk assessment suggests that the probability of appearance of adverse effects derived from chronic exposure to graphene during facemask wearing is extremely low.

## Case 1: silver nanoparticles

### Hazard identification

#### Threshold effects after repeated exposure

Organisation for Economic Cooperation and Development (OECD) (2017) reviewed the repeated dose toxicity studies in animals with silver NPs finding five different studies (studies 1–5 in Table 1). We additionally screened open scientific literature finding two of the studies previously reviewed by OECD (studies 2 and 3 in Table 1) plus two additional studies (studies 6 and 7 in Table 1).

**Table 1:** Summary of repeated dose toxicity studies in rats with silver

Study	Method	Results	Reference
1*	Citrate-capped silver NPs Sprague–Dawley rats 50/sex/dose 62.5, 125 and 250 mg/kg bw/day Gavage 42 days OECD TG 422	No signification toxicity or mortality No significant differences in body weight, food and water consumption in any of the dose groups No statistically significant changes in haematological analysis in any of the treatment groups In the serum biochemical analysis and urinalysis, no treatment related changes No gross or histopathological findings at necropsy NOAEL ≥ 250 mg/kg bw/day	OECD (2017)
2	Citrate-capped silver NPs (60 nm) Sprague–Dawley Rats 10 females/group 30, 300, 1,000 mg/kg/day Gavage 28 days OECD TG 407 GLP	Discharge of mucus granules and an abnormal mucus composition in the goblet cells in the intestines No NOAEL could be derived	Jeong et al. (2010)
3	Citrate-capped silver NPs (60 nm) Fischer 344 rats 10/sex/dose 0, 30, 125 and 500 mg/kg bw/day Gavage 90 days OECD TG 408 GLP	No mortality or clinical signs Decrease in body weight gain in male rats treated with 500 mg/kg bw/day No significant differences in food and water consumption between treated and control groups Significant dose-dependent changes in alkaline phosphatase and cholesterol for males and females, suggesting slight liver damage starting at 125 mg/kg bw/day Histopathologic examination revealed a higher incidence of bile-duct hyperplasia, with or without necrosis, fibrosis, and/or pigmentation starting at 125 mg/kg bw but without clear dose–response twofold increase of silver accumulation in female kidneys compared to male kidneys NOAEL = 30 mg/kg bw/day LOAEL = 125 mg/kg bw/day	Kim et al. (2010) Key study
4*	Citrate-capped silver NPs Sprague–Dawley rats 5/sex/dose 0, 25, 100 and 400 mg/kg bw/day Drinking water 28 days OECD TG 407 non-GLP	No significant toxicity or mortality No significant difference in body weight in any of the dose groups NOAEL ≥ 400 mg/kg bw/day	OECD (2017)
5*	Citrate-capped silver NPs Sprague–Dawley rats 10/sex/dose 0, 25, 100 and 400 mg/kg bw/day Drinking water 90 days OECD TG 408	No significant toxicity or mortality No significant difference in body weight in any of the dose groups NOAEL ≥ 400 mg/kg bw/day	OECD (2017)
6	Polyvinylpyrrolidone-coated silver NPs (14 nm) Wistar rats 2.25 mg/kg bw/day (8 females) 4.5 mg/kg bw/day (8 females) 9 mg/kg bw/day (10 females + 6 males) Gavage 28 days	Clinical, haematological and biochemical parameters, organ weights, macro- and microscopic pathological changes were investigated Faecal bacterial phyla and their silver resistance genes were quantified No toxicological effects NOAEL ≥ 9 mg/kg bw/day	Hadrup and Lam (2014)
7	Silver NPs (7.5 ± 2.5 nm) Pregnant Sprague–Dawley rats 11 dams/dose 0, 100, 300 and 1000 mg/kg bw/day Gavage 14 days (gestation days 6–19)	Dams: oxidative stress in hepatic tissues at ≥ 100 mg/kg/day Foetuses: No teratogenicity or developmental toxicity at doses of up to 1000 mg/kg/day LOAEL adults = 100 mg/kg bw/day NOAEL foetuses = 1000 mg/kg bw/day	Yu et al. (2014) Key study

*NPs*. * = Non-available original study, data extracted from OECD (2017)

Studies 1, 4, 5 and 6 failed in the induction of toxic effects and therefore were not suitable for settings accurate no observed adverse effect level (NOAEL) or lowest observed adverse effect level (LOAEL) (Table 1). The approach of study 2 (Jeong et al. 2010) is histopathological with semi-quantitative end points and, therefore, cannot be used for setting NOAEL or LOAEL.

On the opposite of the above-stated studies, the studies 3 and 7 were able to set NOAEL or LOAEL. We considered these two studies as key studies for estimating a systemic DNEL. It is well known that one of the main clinical effects derived from the silver exposure is argyria (permanent bluish-gray discoloration of the skin or eyes). However, these studies determined that liver is the target of silver NPs. Kim and co-workers (2010) determined in a 90-day exposure study a NOAEL of 30 mg/kg bw/day based on the bile-duct hyperplasia together with slight haematological alterations consistent with hepatic injuries reported at 125 mg/kg bw/day (Table 1). Yu and co-workers (2004) reported in a teratogenicity study in rats maternal hepatotoxicity at 1000 mg/kg bw/day causing oxidative stress in hepatic tissues of pregnant females and set this dose as LOAEL (Table 2). Overall, we consider as key studies for setting systemic DNEL the studies 3 and 7 in Table 1.

**Table 2:** Systemic exposure to silver NPs in silver NP-coated facemask

	General population	Workers
Silver NPs flux (ng/cm^2^/h)	3.8	3.8
Time of exposure (h)	2	8
Exposure surface (cm^2^)	555	555
Pass ng to mg	10^–6^	10^–6^
Body weight	60	60
Systemic exposure (mg/kg bw/day)	7.0 × 10^–5^	2.8 × 10^–4^

Silver NPs flux was taken from the worst case reported by Bianco and co-workers (2014). The rest of parameters were taken as default values considered in risk assessments performed in ECHA and EPA

#### Skin sensitisation

OECD (2017) reviewed two different regulatory studies with citrate capped Ag NPs according to OECD TG 406. In one of these studies, 0.4 ml of Ag NPs of unknown concentration were used for induction and no skin reaction was observed in any of the treated groups 24 and 48 h after the challenge. In the second study, a presumably more severe induction was used (three pairs of intradermal injections of 0.1 ml of 20.48% silver NPs preparation), and 1/20 test animals exhibited grade 1 erythema 24 or 48 h after challenge. Altogether, the available database suggests that Ag NPs could act as weak skin sensitiser.

#### Genotoxicity

The Scientific Committee on Emerging and Newly Identified Health Risks (SCENIHR) (2014) reviewed the health effects and safety of nanosilver. In this opinion, SCENIHR compiled a number of in vitro assays showing positive results in formation of bulky DNA adducts, DNA damage induction, comet assay and micronuclei formation. These positive results were reported at minimum concentrations ranging between units and hundreds of µg/ml. However, negative in vitro results were found in chromosomal aberration test, gene mutation test, micronuclei formation, DNA damage, comet assay in a quite similar range of concentrations. These contradictory assays were explained by SCENIHR based on methodological differences and NP coatings. Nevertheless, it is remarkable that all these effects were identified in open scientific literature and conducted in most of the cases without observing OECD TG and Good Laboratory Practice procedures (GLP). OECD (2017) reviewed one bacterial reverse mutation and one chromosome aberration test both performed observing OECD TG and GLP showing both negative results.

SCENIHR (2014) overviewed an open study showing a weak positive result in *Drosophila melanogaster.* Another comet assay conducted in vivo with leukocytes of Wistar rats after intravenous administration of silver NPs showed a consistent DNA damage. Finally, SCENIHR (2014) also reported negative results in Sprague–Dawley rats in the bone marrow erythrocyte micronucleus test after a 28-day oral exposure and after a 90-day inhalation exposure.

OECD (2017) reviewed two in vivo studies performed according to GLP and conducted following OECD TG 474. In a bone marrow micronucleus assay the oral administration of silver NPs up to 1,000 mg/kg bw/day for 28 days did not increase the incidence of micronuclei formation, suggesting it is not genotoxic under the test conditions. In a second assay, exposure by inhalation up to 2.9 × 10^6^ particles/cm^3^ for 6 h/day, 5 days/week, for 13 weeks did not induce genetic toxicity in male or females. Nevertheless, OECD highlighted that as long as it cannot be demonstrated that the test compound has reached the target tissue, a negative outcome of the test does not guarantee the absence of genotoxicity.

#### Neurotoxicity

The neurotoxicity of silver NPs has been studied in vitro using T98G human glioblastoma cells (Fuster et al. 2020). It was found that the cytotoxicity of silver NPs was low since concentrations of 40 µg/ml were unable to reduce viability of T98G by more than 10% after 72 h of exposure (Fuster et al. 2020). However, other NPs, as zinc and titanium oxides were more cytotoxic to this cell line than silver NPs (Fuster et al. 2021). Silver NPs were unable to incorporate into T98G cells; however, induced transcriptomic alterations indicative of alterations in neuroinflammation processes and in MAPK pathways (Fuster et al. 2020). It suggests that silver NPs can be potentially neurotoxic considering the critical role of glia in the homeostasis of central nervous system.

### Silver NP absorption

#### Oral absorption

We found lack of data for oral absorption of silver NPs in humans. However, it was noted that 18% of silver acetate oral absorption was reported in a 47-year-old woman suffering from argyria associated with the excessive use of an oral anti-smoking remedy containing such substance (East et al. 1980; Hadrup and Lam, 2014). Loeschner and co-workers (2011) studied the distribution of silver in rats following 28 days of repeated oral exposure to silver NPs or silver acetate. They found that 63% ± 23% of silver NP dose was excreted in faeces within a 24-h time period in week 3 of the study, while this record was 49% ± 21% for silver acetate. It suggests that the oral absorption of silver NPs tends to be slightly lower than the ionic silver, although within the same order of magnitude. Therefore, in absence of other more accurate data, we considered for our purposes the record of 18% of oral absorption reported by East and co-workers (1980) for silver NPs.

#### Dermal absorption

We found in the open literature several in vitro studies assessing the dermal absorption of silver NPs in humans and rats. Shape of the silver NPs seems to be a factor that can determine the dermal absorption, although no big differences were found among different forms of silver NPs. The in vitro absorption in rat skin for rod, spherical and triangular silver NPs after 12 h were 1.82, 1.17 and 0.52 µg/cm^2^; respectively (Tak et al. 2015).

Larese and co-workers (2009) demonstrated in an in vitro diffusion cell system the permeability of human damaged skin to 25 nm polyvinylpirrolidone-coated silver NPs. They reported a maximum absorption of 11.6 ng/cm^2^/24 h (approximately 0.48 ng/cm^2^/h). Other studies highlighted that silver percutaneous absorption after exposure to silver NPs depends on the graft sample. The permeability of polyvinylpyrrolidone-coated silver NPs (19 ± 5 nm) after 24-h of silver flux in fresh, cryopreserved and glycerolised skins graft were 0.2, 0.3 and 3.8 ng/cm^2^/h; respectively (Bianco et al. 2014).

There were no big differences among the reported data with human skin and all data ranged between 0.2 and 3.8 ng/cm^2^/h. However, it is noted that silver NPs absorption in rats seems to be higher (in the order of 150 ng/cm^2^/h) than in humans. In the name of worst realistic approach, we will consider the value reported by Bianco and co-workers (2014) of 3.8 ng/cm^2^/h as key value for our calculations.

### Systemic exposure

The repeated dose toxicity studies summarised in Table 1 employ in all cases oral route. The exposure to silver NPs via facemask wearing is obviously dermal. Thus, to be able to compare critical end points of the key studies shown in Table 1 with the real exposure we need to estimate the systemic (internal) exposure. For such purpose, we used the following equation:

$$Systemic\;exposure\;\left( {mg/kg \, bw/day} \right)\; = \;Flux \, \left( {mg/cm^{2} /h} \right) \, \times \, surface\; \, of\; \, exposure \, \left( {cm^{2} } \right) \, \times \;{{exposure\;time\;\left( h \right)} \mathord{\left/ {\vphantom {{exposure\;time\;\left( h \right)} {body\;weight}}} \right. \kern-\nulldelimiterspace} {body\;weight}}.$$

As commented above as reference value for influx the worst case described by Bianco and co-workers (2014) of 3.8 ng/cm^2^/h will be considered. We will consider for general population and workers 2 and 8 h of daily exposure; respectively. As regard area of exposure, we are going to consider 555 cm^2^. This figure is taken from the recommendations of default human factor values for use in exposure assessment for biocidal products used by the European Chemical Agency (ECHA) that refer to US-EPA Human Factors Handbook (2011a). The 555 cm^2^ value assumes that the whole surface of the face will be covered by the mask. We will use 60 kg body weight as well according to the same recommendation (US-EPA, 2011b). The estimation of systemic exposure is described in Table 2. The estimated values for general population and workers were 7 × 10^–5^ and 2.8 × 10^–4^ mg/kg bw/day; respectively.

### Systemic DNEL estimation

As commented above the exposure to silver NPs through facemask wearing takes place via a different route from those used in the critical end points obtained in Table 1 and, therefore, to compare a derived no effect level (DNEL) with the dermal exposure, we need to use systemic exposures as derived in Table 2 with systemic DNELs. For these DNELs estimation we will use as endpoints the NOAEL and LOAEL obtained in the 90-day oral toxicity study (Kim et al. 2010) (study 3 in Table 1) and the LOAEL obtained for pregnant dams in the teratogenicity study in rats (Yu et al. 2014) (study 7 in Table 1). Other assessment factors were considered according to the ECHA procedures for deriving DNELs for threshold endpoints (ECHA, 2012).

Table 3 shows the results of the systemic DNEL estimation. The endpoint that yielded the highest DNEL was the NOAEL of the 90-day toxicity study, while the LOAEL of the teratogenicity study yielded the lowest DNEL. Estimated systemic DNELs ranged between 0.01 and 0.0375 mg/kg bw/day for general population and between 0.02 and 0.075 mg/kg bw/day for workers.

**Table 3:** Estimation of systemic DNEL for silver NPs

	NOAEL (Kim et al. 2010)		LOAEL (Kim et al. 2010)		LOAEL (Yu et al. 2014)	
	GP	W	GP	W	GP	W
Critical value (mg/kg bw/day)	30	30	125	125	100	100
Oral absorption	0.18	0.18	0.18	0.18	0.18	0.18
Assessment factors						
Interspecies allometric factor	4	4	4	4	4	4
Interspecies remaining factor	2.5	2.5	2.5	2.5	2.5	2.5
Intra-species factor	10	5	10	5	10	5
LOAEL to NOAEL	1	1	3	3	3	3
Adjustment to chronic	2	2	2	2	6	6
Systemic DNEL (mg/kg bw/day)	0.027	0.054	0.0375	0.075	0.010	0.020

Critical values were taken from the referred studies summarised in Table 1. Oral absorption was taken from East et al. (1980). Assessment factors were set according to the ECHA procedures for deriving DNELs for threshold endpoints (ECHA 2012). The duration of the teratogenicity study (Yu et al. 2014) was considered sub-acute. *GP* = General population, *W* = Workers

### Risk characterisation

#### Threshold effects after repeated exposure

The risk characterisation ratio (RCR) was estimated as the ratio between systemic exposure and the systemic DNEL. Results are shown in Table 4. The RCR estimated for general population was 0.007; while for workers the record was of 0.014.

**Table 4:** Risk characterisation for dermal exposure to silver NP-coated facemasks

	Exposure (mg/kg bw/day)	DNELb (mg/kg bw/day)	RCR
General population	7.0 × 10^–5^	0.010	0.007
Workers	2.8 × 10^–4^	0.020	0.014

Exposure was extracted from Table 2. In a conservative approach the lowest DNEL estimated in Table 3 were extracted for estimation of risk characterisation ratio (RCR)

As commented above certain neurotoxic effects were reported in vitro after exposure of T98G human glioblastoma cells to silver NPs. The authors reported in this study 0.5 µg/ml the lowest silver NPs concentration able to cause a detectable effect in transcription of certain genes (Fuster et al. 2020). Ministry of Health of Canada Government (2013) reported that the highest (the upper edge of the 95% confidence interval of the 95th percentile) silver concentration circulating in whole blood in the Canadian population was 0.42 μg/l (42 ng/ml). This concentration is 2 orders of magnitude than the concentration reported as neurotoxic in vitro and very far from those concentrations that could be reached after facemask wearing.

#### Skin sensitisation

The information found suggests that silver NPs could be a weak skin sensitiser. However, as stated above only 5% of animals were mildly sensitised (erythema score 1 after 48 h of challenging) in a regulatory study; while a second study failed demonstrating skin sensitisation power of silver NPs.

The amount of silver NPs intradermally injected to induce skin sensitisation in the positive study was around 60 mg (3 injections of 0.1 ml of a 20.48% preparation). Considering the worst exposure estimated in Table 2 (2.8 × 10^–4^ mg/kg bw/day) the number of days needed to reach 60 mg of silver NPs from facemasks wearing would be around 3500 (assuming no elimination). Altogether, this suggests that, given the weak sensitising potential of silver NPs, the risk of skin sensitisation derived from wearing silver NPs coated facemasks is negligible. This is also supported by the fact that, approximately after 1.5 years of pandemic, no epidemiological studies reporting skins sensitisation were found in the open scientific literature.

#### Genotoxicity

The database provides an array of in vitro studies showing positive results. However, other in vitro studies contradicted these positive results. Moreover, regulatory in vivo studies did not obtain positive results. Even in the case that the positive results in vivo were not reproduced due to lack of accessibility of silver NPs to bone marrow, the positive in vitro results were obtained using silver concentration that are not physiologically feasible (µg/ml). Overall, the genotoxicity of silver NPs is still doubtful and more research as this regard is needed, although the in vitro available information suggests that the risk, if any, should be very low.

### Discussion of case 1

We have estimated the risk of wearing silver NPs facemasks as regard as threshold effects, skin sensitisation and genotoxicity. The results presented in this work suggest that the risk of skin sensitisation is negligible; while the risk for genotoxicity is very low. The risk for threshold effects is also very low; specifically, the exposure is 143 times lower than needed to cause hepatotoxicity in general population and 71 times in workers.

Our assessment describing the safety of wearing silver NPs coated masks is supported by the provisional tolerable intake derived for intravenous exposure to silver NPs released from medical devices. This provisional tolerable intake is 0.14 µg/kg bw/day (Savery et al. 2017), therefore in the same order of magnitude that the systemic exposure obtained in Table 2.

Our assessment for threshold effects has uncertainties. However, we consider that the estimated risk is overestimated because it has been suggested that silver NPs are indeed able to penetrate in vivo the *stratrum corneum* and reach reticular dermis, although silver NPs are deposited into the dermis and do not reach systemic circulation (George et al. 2014). This can be deduced from a study where a nanocrystalline silver dressing was applied to a sample of 16 healthy patients with normal intact skin and approximately after 5 days of exposure no increase in silver blood circulating could be detected (George et al. 2014).

We also estimated that our assessment is very conservative by a second reason. We used for setting the systemic DNEL the NOAEL and LOAEL considered in each of the key studies shown in Table 1. These records are considered as very conservative. Kim and co-workers (2010) set a LOAEL based on mild haematological alterations suggesting hepatic impairments and bile-duct hyperplasia (with or without necrosis), fibrosis, and pigmentation. However, it is noted that the dose–response of these histopathological findings is unclear and therefore the liver impairment questionable. In addition, the teratogenicity study in rats (Yu et al. 2014) set a LOAEL based on oxidative stress in hepatic tissues; which could be also interpreted as an adaptive reaction to silver exposure rather than a liver malfunction at this exposure level. Thus, in none of these two key studies, a real liver impairment based on robust histopathological findings was consistently demonstrated.

## Case 2: graphene nanoparticles

### Hazard identification

#### Threshold effects

Our bibliographic search about adverse effects induced by graphene after repeated exposure reported six different studies (Table 5). As regard the route of exposure of these studies, one used intraperitoneal administration, two used inhalation exposure, two used gavage and one used drinking water as route of administration. Only one of these studies was considered with regulatory applicability (study 3 in Table 5).

**Table 5:** Summary of repeated dose toxicity studies in rodents with graphene NPs

Study	Method	Results	Reference
1	GQD-PEG (10–30 µm wide and 0.5–2 nm in height) Balb/c mice 12 females/group 20 mg/kg/day Intraperitoneal injection 14 days	3/12 mortalities (days 4, 5 and 6) Died without any clinical signs Animals with dark livers and spleens with dark spots with tens of micrometers in diameter (presumably GQD-PEG bioaccumulation) Blood biochemistry and haematology suggest no obvious toxicity of GQD-PEG	Chong et al. 2014
2	Graphene oxide nanopowder (thickness 1 ~ 2 atom layer) Sprague–Dawley rats 15 males/group 0, 0.76, 2.60 and 9.78 mg/m^3^ Nose-only inhalation exposure system 6 h/day 5 days Recovery for 1, 3, and 21 days	No significant body or organ weight changes No effects on: blood biochemistry and haematology, bronchoalveolar lavage fluid inflammatory markers No effects on bronchoalveolar lavage fluid lymphocytes, macrophages, or polymorphonuclear cells Spontaneous clearance of graphene oxide-ingested alveolar macrophages No histopathological lesions in liver and kidneys	Kim et al. 2018
3	Graphene platelets (thicknesses, ranging from 0.350 to 0.380 nm, 96% carbon, 4% oxygen, 750 m^2^/g surface area of particles, 0.2 g/ml, < 2 µm average lateral dimension, 20–30 layers average thickness) Nominal concentrations: 0, 0.12, 0.47 and 1.88 mg/m^3^ Mass median aerodynamic diameter: 123 ± 3 nm Inhalation Sprague–Dawley rats OECD TG 412 28-days	No clinical signs No mortality Significant body weight losses: 0.47 mg/m^3^ at week 2 and 1.88 mg/m^3^ at weeks 1, 5, 6, 11, and 13 weeks No toxicologically relevant or concentration-related haematological alterations No concentration-related effects in the inflammatory or oxidative stress biomarkers in the bronchoalveolar lavage fluid No significant alterations in levels of various cytokines In bronchoalveolar lavage fluid the total cell counts and macrophage counts were significantly decreased in all the exposed groups at the 1-day post-exposure and 28-day post-exposure Significant thymus weight loss and brain weight gain at the top dose No gross pathological findings The ingested graphene in the lung macrophages persisted even after the 90-day post-exposure period	Kim et al. 2016*
4	Graphene oxide (40 nm diameter, zeta potential value = -33.2 mV) Sprague–Dawley rats 5 males/group 0, 10, 20 and 40 mg/Kg bw/day 5 days Gavage	Dose-dependent increase of the superoxide dismutase, catalase and glutathione peroxidase activities in kidneys Increase in serum creatinine and blood urea nitrogen levels Significant elevation in the levels of lipid hydro peroxide Significant histopathological alterations (progressive dilation of tubules, tubular necrosis, renal tubular separation, degeneration of hematopoietic tissue and tubular lumen) in kidneys	Patlolla et al. 2016
5	Reduced graphene oxide nanosheets (~ 25 mm) C57black/6 mice 5 males/group 60 mg/kg body weight 5 days Gavage 1, 15 and 60 days recovery	Open field test: No effect on the exploratory and anxiety-like behaviours within 60 days of the final treatment Rotarod test: On day 1 physical decline and decreased neuromuscular coordination; no effects on days 15 and 60 Morris water maze test: Mouse learning and memory not affected at any time No effects on liver and kidney functions and haematology values No dysfunction in hippocampus	Zhang et al. 2015
6	Graphite oxide (carbon/oxygen molar ratio = 2.1 ICR mice 6 pups/group 0, 0.05 and 0.5 mg/ml Drinking water Dose during post-natal days 1–38 Observation at post-natal days 21 and 38	No difference in behaviours 0.5 mg/ml: Retarded increase of body weight, body length and tail length No effects on blood biochemical indicators on kidney and liver No morphological changes in lung, spleen, heart and kidney at any dose and any time Decreased villus length of duodenum at day 38 of 0.5 mg/ml group	Fu et al. 2015

* = Original study non-available to authors, data extracted from Graphene REACH registration dossier available on October 6 in https://echa.europa.eu/es/registration-dossier/-/registered-dossier/24678

In the intraperitoneal study (study 1 in Table 5) (Chong et al. 2014) it was noted that 20 mg of polyethylene glycol graphene quantum (GQD-PEG) caused 25% of mortality (3/12 female Balb/c mice) without relevant alterations other than the darkening of liver and spleen. This allowed the authors to speculate that animals died by graphene bioaccumulation. However, this explanation is discussable since mortalities occurred on days 4, 5 and 6 of exposure and no other dead were reported between day 7 and 14 of exposure.

In a non-regulatory study, Kim and co-workers (2018) (study 2 in Table 5) were unable to detect toxicity in rats exposed up to 9.78 mg/m^3^ of graphene oxide nanopowder during 5 days (6 h/day). Similarly, non-significant toxicological effects were reported in a 28-day inhalation study performed observing OECD TG 412 in which rats were daily exposed during 6 h up to 1.88 mg/m^3^ of graphene nanoplatelets (Graphene REACH Registration dossier) (study 3 in Table 5).

Patlolla and co-workers (2016) (study 4 in Table 5) described kidney as target organ of graphene oxide after dosing rats by gavage during 5 days with up to 40 mg/kg bw/day. Indeed, this exposure regime induced haematological alterations together with severe histopathological alterations. On the opposite, Zhang and co-workers (2015) (study 5 in Table 6) failed to induce nephrotoxicity in mice treated by gavage with reduced graphene with 60 mg/kg bw/day. It is noted that, the graphene preparations used in studies 4 and 5 of Table 5 were different, since in the first case it as graphene oxide; while in the second it was reduced graphene and this difference could explain why Zhang and co-workers (2015) did not induce kidney alterations with a dose higher than Patlolla and co-workers (2016) did.

**Table 6:** Summary table of in vitro and in vivo mutagenicity/genotoxicity studies with graphene NPs

Stydy	Method	Concentrations	Results	Reference
1	In vitro cytogenicity/chromosome aberration study in mammalian cells OECD TG 473 Primary cultures of human peripheral blood lymphocytes Donors: non-smoking males, 18–35 years	Graphene platelets (thicknesses, ranging from 0.350 to 0.380 nm, 96% carbon, 4% oxygen, 750 m2/g surface area of particles, 0.2 g/ml, < 2 µm average lateral dimension, 20–30 layers average thickness) 0, 20.0, 63.2, 200, 632, 1000 µg/ml Maximal concentration recommended by the guideline (2000 µg/ml) caused heavy precipitate/insoluble material Positive control substance: Cyclophosphamide and mitomycin C With and without exogenous metabolic activation system	Negative control: Within the 95%, control limits of the distribution of the negative control database Positive controls: Statistically significant increases in the number of aberrant cells compared with the concurrent negative controls Graphene: No statistically significant increases in the proportion of aberrant metaphases at any experimental point	Graphene REACH registration dossier
2	In vitro comet assay BEAS-2B human bronchial epithelial cells	5 different graphene forms: pristine, carboxylated nanoplatelets, aminated nanoplatelets, single layer and few layer graphene oxide 0, 10 and 50 mg/ml 24 h	10 mg/ml reduced cell viability by less than 10% in all forms 50 mg/ml reduced cell viability by around 20% in all isoforms All graphene nanoforms were positive at 50 mg/ml but not at 10 mg/ml	Chatterjee et al. 2016
3	In vitro comet assay Human lung fibroblast	Graphite oxide flakes, polyethylenimine functionalized graphene oxide, polyethylene glycol functionalized graphene oxide, lactobionic acid-polyethylene glycol functionalized graphene oxide 0, 1, 50 and 100 mg/ml 24 h	Graphite oxide flakes, polyethylenimine functionalized graphene oxide and polyethylene glycol functionalized graphene oxide were positive at concentrations in which graphite oxide flakes were notably cytotoxic Lactobionic acid-polyethylene glycol functionalized graphene oxide were negative	Wang et al. 2013
4	In vitro comet assay Chromosomal aberrations Human mesenchymal stem cells from umbilical cord blood	Reduced graphene oxide of different size of particles 0, 0.01, 0.1, 1, 10, 100 µg/ml 24 h	Weak positive results for both comet and chromosomal aberrations starting at 0.1 µg/ml depending on size of particle Cytotoxicity was noted starting by 1 µg/ml	Akhavan et al. 2012
5	In vitro comet assay NIH-3T3 mouse fibroblast cells A549 human lung carcinoma cells MDA-MB-231 human breast adenocarcinoma cells	N-doped graphene quantum dots (10.9 nm diameter) 50, 100 and 150 mg/ml	70% cell viability at 200 mg/ml for A549 and MDA 70% cell viability at 120 mg/ml for NIH-3T3 Statistically significant genotoxic damage at the 100 and 150 mg/ml doses A549 cells more resistant to genotoxicity than NIH-3T3 and MDA-MB-231	Şenel et al. 2019
6	Comet assay BALB/c mice spermatogonial stem cells	Graphene oxide and reduced graphene oxide 1, 10, 100 and 400 µg/ml	Graphene oxide induced cell death at 100 and 400 µg/ml (both concentrations by around 40%) Reduced graphene oxide induced a cell death at 400 µg/ml (by around 40%) Graphene oxide induced significant DNA fragmentation at 100 and 400 µg/ml Reduced graphene oxide did not induce significant DNA fragmentation	Hashemi et al. 2016
7	Mammalian erythrocyte micronucleus test OECD TG 474 Sprague–Dawley rats 5/sex/dose	Graphene platelets (thicknesses, ranging from 0.350 to 0.380 nm, 96% carbon, 4% oxygen, 750 m2/g surface area of particles, 0.2 g/ml, < 2 µm average lateral dimension, 20–30 layers average thickness) Inhalation 0, 0.5, 1, 2 mg/L air 3 days for up to 240 min Individual mass median aerodynamic diameter: 2.9–5.2 µm Positive control: cyclophosphamide (20 mg/kg)	No mortalities No clinical signs Reductions in body weight gain at the top dose Positive control: clear and unequivocal increase in micronuclei Graphene: No statistically significant increases in the incidence of micronucleated polychromatic erythrocytes	Graphene REACH registration dossier

In a study with developing pups retardation in increase of body weight, body length and tail length were attributed to the graphene oxide exposure via drinking water. The authors attributed these alterations to noted decreased villus length of duodenum that could be reducing the efficiency of nutrient absorption (Fu et al. 2015) (study 7 in Table 5). On the contrary, in study 6 (Table 5) with graphene oxide, no nephrotoxicity was reported.

In addition to the above summarised and described repeated dose toxicity studies our bibliographic search also found some acute toxicity studies with graphene. Li and co-workers (2013) intratracheally instilled C57BL/6 mice with 0, 1, 5 and 10 mg/kg of nanoscale graphene oxide finding substance aggregation followed by pulmonary inflammatory response, pulmonary parenchymal oedema, acute lung injury and chronic pulmonary fibrosis. In another intratracheal instillation study 5 and 50 µg of few layer graphene (carbon/oxygen molar ratio = 89:6) were administered to mice (6 animals/group) and 24 h after instillation in the 50 µg group inflammatory cell infiltration together with lung cell injury were noted (Mao et al. 2016). Lungs of animals dosed with 50 µg of few layer graphene turned black and showed mild to moderate interstitial oedema and parenchymal oedema together with the presence of multiple lung macrophages in the alveolis (Mao et al. 2016).

The literature also shows several in vitro toxicity studies with graphene. In this sense, concentrations up to 160 µg/ml of GQD-PEG did not induce after 24 h of exposure alterations in membrane integrity or mitochondrial function of HeLa cells and were also unable to induce oxidative stress and apoptosis (Chong et al. 2014). Nevertheless, Achawi and co-workers (2021) found after a systemic literature review considering 185 graphene NMs that there is a large variety of cytotoxic effects and that, with the available information, it is not possible to establish a clear structure–activity relationship for most of the materials due to their poor physical characterisation and the variety of biological end points employed in in vitro toxicity studies.

In summary, as usual, the specific nanoform, seems to play a pivotal role in the toxicity of the NP. The reported adverse effects in the assessed studies were dependent on route of exposure. Only after acute intratracheal instillation of graphene NMs severe pulmonary effects (pulmonary inflammatory response, pulmonary parenchymal oedema, chronic pulmonary fibrosis, inflammation) were reported; while the inhalation studies failed to reproduce such adverse effects; which suggests that pulmonary absorption could be notably low. The oral route studies also reported nephrotoxicity for same graphene forms. Finally, it is noted that no studies of toxicity using dermal route (the most relevant route for the exposure via facemasks) were found.

#### Skin sensitisation

The Graphene REACH registration dossier presents a skin sensitisation study conducted observing OECD TG 406. This REACH registration dossier was presented for graphene platelets with thicknesses ranging from 0.350 to 0.380 nm, a carbon/oxygen ratio 96/4, a surface area of 750 m^2^/g, a density of 0.2 g/ml, an average lateral dimension < 2 µm and an average thickness of 20–30 layers. In this study, twenty male Hartley guinea pigs were induced 6 h/week for 3 weeks (days 1, 8, 15) with 0.9% saline + 0.25 g graphene and on day 29 challenged during 6 h with 0.9% saline + 0.25 g graphene. In all cases, the exposure was epicutaneous with occlusive wrap. In parallel, control group and positive control groups (10 animals each) were induced with saline and challenged with 0.9% saline + 0.25 g graphene or 0.4 ml α-hexylcinnamaldehyde. There were no test substance-related clinical observations or body weights alterations. No indication of skin sensitisation was noted neither 24 nor 48 h after challenge in animals induced with graphene; while animals induced with α-hexylcinnamaldehyde showed positive indication of skin sensitisation. Thus, graphene is not a skin sensitiser under the conditions described in this study.

Kim and co-workers (2021) studied the skin sensitising potential of a graphene preparation (diameter < 2 microns, and a thickness of a few nm and surface area of 300 m^2^/g) using an in vitro assay (KeratinoSens™ Assay) and an alternative in vivo method (local lymph node assay with BALB/C mice). In the first in vitro test the EC1.5 (interpolated concentration for a 1.5-fold luciferase induction) value for the graphene NPs was higher than 2000 µM; which allows considering the preparation as a non-sensitizer. This result was confirmed in the local lymph node assay since the reported stimulation index was lower than three. Thus, the results of both tests support the above commented regulatory test and highlight again the fact that graphene is not a sensitising agent.

#### Genotoxicity

Our search about graphene genotoxicity yielded seven studies, six of them in vitro (one chromosome aberration study in mammalian cells plus five comet assays) and only one (a mammalian erythrocyte micronucleus test) in vivo. Only two of them were performed observing OECD Guidelines. These studies were summarised in Table 6.

The in vitro cytogenicity/chromosome aberration study in human peripheral blood lymphocytes (study 1 in Table 6) was performed observing OECD TG 473 and yielded a negative result. However, remaining in vitro comet studies shown in Table 6 (studies 2–6) with BEAS-2B human bronchial epithelial cells, human lung fibroblast cells, human mesenchymal stem cells from umbilical cord blood, mice spermatogonial stem cells and different human carcinoma cells yielded positive results. It is remarkable that some of these in vitro positive results were obtained at borderline concentrations to excessive cytotoxicity (studies 2, 4 and 5) or even with severe cytotoxicity (studies 3 and 6); which diminish the concern in front of a physiologically relevant exposure.

The in vivo mammalian erythrocyte micronucleus test in rats performed observing OECD TG 474 failed to detect statistically significant increase in the incidence of micronucleated polychromatic erythrocytes. Therefore, the positive in vitro results could not be confirmed by a regulatory study in vivo.

### Graphene absorption

The relevant route of exposure in the case of facemasks wearing is dermal. However, no toxicological studies by dermal route were found in Table 5. Thus, as in the case of silver NPs, it is necessary to estimate the systemic (internal) exposure, and for such purpose, the absorption rates for different routes of exposure have to be considered.

#### Oral absorption

The oral route is not relevant for NPs exposure and we did not find robust information at this regard coming from standard toxicokinetic studies. Nevertheless, study 4 in Table 5 (Patlolla et al. 2016) showed, as graphene oxide is able to induce severe nephrotoxicity after gavage; which is a clear indicative that the NP has been absorbed to some extent. Zhang and co-workers (2015) concluded that reduced graphene oxide labelled with ^125^I was able to reach the main body organs after oral administration, although the percentage of absorption was not quantified in this study. However, this result is not supported by the findings reported by Yang and co-workers (2013); who noted that ^125^I labelled polyethylene glycolated graphene oxide was not obviously incorporated in tissues after oral administration, indicating the rather limited intestinal adsorption of these NMs. In conclusion, the oral absorption rate could not be deduced from the available database.

#### Inhalation absorption

The inhalation repeated dose toxicity studies summarised in Table 5 show no obvious adverse effects; which points towards a low absorption by inhalation. However, approximately 15% of ^125^I-polyethylene glycolated graphene oxide intratracheally instilled in mice was excreted in 6 h in urine indicating that the NM can penetrate the alveolar–capillary barrier into blood and be quickly eliminated by a renal route (Li et al. 2013). This study did not quantify the absorption, but Mao and co-workers (2016) confirmed the above-stated facts about the lack of oral absorption and estimated that 47% of few layer graphene labelled with ^14^C remained in the lungs 4 weeks after intratracheal instillation.

#### Dermal exposure

Poland and co-workers (2013) reviewed the dermal absorption of NMs and concluded that there are conflicting results but, in general, the dermal absorption of any NM is theoretically feasible, although in low degree, especially for those NMs of large size, for which dermal absorption should be lower than those NMs of lower size.

We were unable to find studies about graphene dermal absorption. The REACH Registration dossier for graphene does not contain a dermal absorption study. However, this dossier presents an in vitro skin irritation study with reconstructed human epidermis. In this study, few layer graphene was found aggregated/agglomerated in the epidermis surface after 42 min of semi occlusive exposure. This study also reported few layer graphene depots smaller than the aggregates/agglomerates observed above the epidermis within the *stratum corneum* but not in inner skin layers. It suggested to the graphene registrant that the NM is not able to pass through *stratum granulosum*, *stratum spinosum* and *stratum basale* and therefore reach systemic circulation.

In conclusion, there is no relevant information about the graphene dermal absorption; although the limited available information suggests that the absorption should be rather low. This is also confirmed by Ou and co-workers (2016) when concluding that there is insufficient evidence available to conclude that graphene can penetrate skin. This is also supported by the fact that no information about dermal absorption of other carbon based NMs as single or multiple wall carbon nanotubes is available and by the fact that there is no indication that nanocarbon black (particle size < 40 nm) used in cosmetics were absorbed through intact skin when measured with an imaging method (SCCS 2013).

### Systemic exposure

Due to the above-stated unavailability of information about dermal absorption, it is concluded that it is not possible to derive a systemic exposure to graphene after dermal exposure derived from wearing facemasks coated with graphene.

### Systemic DNEL estimation

The approach followed in case 1 is unfeasible in the case of graphene due to the impossibility to estimate a systemic exposure and also due to the impossibility to estimate a systemic DNEL due to lack of reliable information about absorption after inhalation or oral exposures. Thus, a non-quantitative risk assessment has been considered as appropriate.

### Risk characterisation

#### Threshold effects after repeated exposures

The possibility of adverse effects after repeated dermal exposures cannot be totally ruled out due to the uncertainties addressed to dermal absorption, although the available information suggests that such dermal absorption, if any, should be very low and consequently, the adverse effects should be very unlikely.

#### Skin sensitisation

One regulatory assay performed observing OECD TG 406, one KeratinoSensTM in vitro test and one local lymph node assay yielded negative results when the tested substance was graphene. Thus, graphene is not a skin sensitiser and the dermal exposure derived from wearing graphene coated facemask does not pose a risk of skin sensitisation for both general population and workers.

#### Genotoxicity

Table 6 summarise the database for genotoxicity. Two regulatory (one in vivo and one in vitro) tests were negative, although an array of in vitro tests showed a few positive results sometimes in conditions producing cytotoxicity. The Guidance on the Application of the Classification, Labelling and Packaging Criteria (2017) published by ECHA establishes that a substance causes concerns of mutagenicity when there is positive evidence obtained from somatic cell mutagenicity tests in vivo, in mammals; or other in vivo somatic cell genotoxicity tests which are supported by positive results from in vitro mutagenicity assays. None of these conditions has been met for graphene and therefore the risk of genotoxicity derived from dermal exposure should not be considered a concern.

### Discussion of case 2

We have detected important and relevant gaps in the database that precludes the possibility of a risk assessment with a reasonable uncertainty. These gaps affect mainly to dermal absorption that, although it seems it should be low, should be accurately determined.

The regulatory skin sensitisation test performed according to OECD TG was negative. However, it is noted that in this assay the induction phase was performed with dermal exposure instead of intradermal induction as usual. Considering the presumably low skin absorption, the result of this test flags some information gaps in terms of hazard identification, although the negative results in the in vitro and local lymph node assays reduce these concerns. Nevertheless, this uncertainty in hazard identification does not alter the result of the risk assessment that is maintained negligible due to the limited dermal absorption.

## General conclusions

The risk assessment presented for both NMs suggests that, despite the uncertainties, the risk of adverse threshold, non-threshold and skin sensitisation effects derived from the wearing of silver or graphene coated NP face masks is low or even negligible. Therefore, the use of this protection device should be strongly encouraged to workers and general population as tool of defence against COVID-19.

We have reviewed the labels of several face masks coated with NMs and in none of them the amount and characteristic of the NM was available. The adverse effects of NMs depend, among other factors, of their physical properties. Thus, to perform further tier assessments, the physical characteristics of the coating NMs should be publically available. This would allow the regulatory agencies to conduct more refined risks assessments of these materials; thus, ensuring a safe use of this type of facemasks for consumer use.

## Footnotes

1 **Abbreviations:** CLP = Classification, Labelling and Packaging; DNEL = Derived systemic No Effect Level; ECHA = European Chemical Agency; GLP = Good Laboratory Practice; GQD-PEG = Polyethylene glycol graphene quantum; LOAEL = Lowest Observed Adverse Effect Level; NMs = Nanomaterials; NOAEL = No Observed Adverse Effect Level; NPs = Nanoparticles; OECD = Organisation for Economic Cooperation and Development; RCR = Risk Characterisation Ratio; SCCS = Scientific Committee on Consumer Safety; SCENIHR = Scientific Committee on Emerging and Newly Identified Health Risks; WHO = World Health Organization.

2 https://www.who.int/emergencies/diseases/novel-coronavirus-2019/events-as-they-happen (accessed last time on October 6, 2021).

3 https://healthycanadians.gc.ca/recall-alert-rappel-avis/hc-sc/2021/75309a-eng.php (accessed last time on October 6, 2021).
